# Twitter Discussions on #digitaldementia: Content and Sentiment Analysis

**DOI:** 10.2196/59546

**Published:** 2024-07-16

**Authors:** Hyeongchan Cho, Kyu-Min Kim, Jee-Young Kim, Bo-Young Youn

**Affiliations:** 1 Department of Business Administration Graduate School Kyung Hee University Seoul Republic of Korea; 2 Department of Health Administration Gyeonggi University of Science and Technology Gyeonggi-do Republic of Korea; 3 Medical R&D Center Bodyfriend Co Ltd Seoul Republic of Korea; 4 Department of Bio-Healthcare Hwasung Medi-Science University Gyeonggi-do Republic of Korea

**Keywords:** digital dementia, dementia, public health, Twitter, social media, mobile phone

## Abstract

**Background:**

Digital dementia is a term that describes a possible decline in cognitive abilities, especially memory, attributed to the excessive use of digital technology such as smartphones, computers, and tablets. This concept has gained popularity in public discourse and media lately. With the increasing use of social media platforms such as Twitter (subsequently rebranded as X), discussions about digital dementia have become more widespread, which offer a rich source of information to understand public perceptions, concerns, and sentiments regarding this phenomenon.

**Objective:**

The aim of this research was to delve into a comprehensive content and sentiment analysis of Twitter discussions regarding digital dementia using the hashtag #digitaldementia.

**Methods:**

Retrospectively, publicly available English-language tweets with hashtag combinations related to the topic of digital dementia were extracted from Twitter. The tweets were collected over a period of 15 years, from January 1, 2008, to December 31, 2022. Content analysis was used to identify major themes within the tweets, and sentiment analysis was conducted to understand the positive and negative emotions associated with these themes in order to gain a better understanding of the issues surrounding digital dementia. A one-way ANOVA was performed to gather detailed statistical insights regarding the selected tweets from influencers within each theme.

**Results:**

This study was conducted on 26,290 tweets over 15 years by 5123 Twitter users, mostly female users in the United States. The influencers had followers ranging from 20,000 to 1,195,000 and an average of 214,878 subscribers. The study identified four themes regarding digital dementia after analyzing tweet content: (1) cognitive decline, (2) digital dependency, (3) technology overload, and (4) coping strategies. Categorized according to Glaser and Strauss’s classifications, most tweets (14,492/26,290, 55.12%) fell under the categories of wretched (purely negative) or bad (mostly negative). However, only a small proportion of tweets (3122/26,290, 11.86%) were classified as great (purely positive) or swell sentiment (mostly positive). The ANOVA results showed significant differences in mean sentiment scores among the themes (*F*_3,3581_=29.03; *P*<.001). The mean sentiment score was –0.1072 (SD 0.4276).

**Conclusions:**

Various negative tweets have raised concerns about the link between excessive use of digital devices and cognitive decline, often known as digital dementia. Of particular concern is the rapid increase in digital device use. However, some positive tweets have suggested coping strategies. Engaging in digital detox activities, such as increasing physical exercise and participating in yoga and meditation, could potentially help prevent cognitive decline.

## Introduction

### Background

Excessive dependence on electronic devices such as the internet or smartphones can cause digital dementia, that is, cognitive impairment such as decreased attention or memory and, more seriously, promote the early onset of dementia [1]. According to a meta-analysis of 74 studies encompassing 2.8 million adults aged 30 to 64 years, 119 per 100,000 people develop young-onset dementia, amounting to 3.9 million cases worldwide; as a result, Alzheimer disease was found to be the most prevalent, followed by vascular and frontotemporal dementias [2]. Furthermore, the number of people with dementia is also estimated to nearly triple to >152 million by 2050 [3].

The term *digital dementia* was first introduced by the German neuroscientist Manfred Spitzer in 2012, suggesting that excessive use of digital technology can lead to cognitive decline and deterioration of short-term memory [4]. The widespread adoption of various digital screen-based media, including watching television; using the internet; texting; and using social media platforms such as Instagram, Facebook, and Twitter (subsequently rebranded as X), can impair attention and memory recall, which is a growing concern globally [5,6].

Excessive use of the internet can have long-term negative effects [7]. It can impact the brain’s ability to process visual information and decrease concentration [8]. Furthermore, it has been linked to various mental health disorders and structural changes in the brain [9]. In addition, it can significantly impair cognitive function, attention control, decision-making processes, and working memory [10].

The COVID-19 pandemic has led to a rapid increase in the frequency of digital technology use. Unfortunately, this increased dependence on digital devices has resulted in physical inactivity, emotional instability, sleep disorders, and memory impairment [11-14]. Studies have shown that the mere presence of a smartphone can have negative effects on cognitive function, resulting in a reduced capacity of attention and impaired task performance [15-17]. Even when the device is not being consciously used, it can still impact cognitive ability [18].

The negative impact of the increased use of digital technology on mental health has been a topic of interest for researchers [19,20]. It can increase symptoms of attention-deficit/hyperactivity disorder, interfere with emotional and social intelligence, lead to addictive behavior, and contribute to social isolation; additionally, it can disrupt cognitive and brain development, affect sleep patterns, and have other adverse effects [8]. Furthermore, factors such as dependence on technology, information overload, and excessive screen time can harm brain health and cognitive capabilities, increasing the risk of Alzheimer disease [21]. Certain groups are at a high risk of experiencing digital dementia, particularly children and adolescents, whose brains are not yet fully matured and who rely heavily on electronic devices [22]. Overuse of such devices at a young age can lead to symptoms of cognitive impairment, including short-term memory loss, and developmental delay [23]. It is important to be aware of these risks and take measures to prevent overuse of electronic devices in young individuals [24,25]. Extensive research in the field of biopsychosocial studies has provided evidence that using digital devices excessively during crucial periods of brain development, for >2-3 hours a day, can have detrimental effects on individuals. These effects include impairments in learning and memory, attention and emotional disorders, substance use disorder, and negative changes in neurological disorders [26].

Given the rapidly increased frequency of the use of social media, it is vital to consider the potential of social media, also known as digital data, in public health surveillance and prevention. There have been promising developments of hybrid approaches that combine traditional surveillance data with digital data, which often capture the accurate spreading of information in a timelier manner [27]. Social media is known for the rapid identification of an outbreak of infectious diseases [28]. A study reported the prediction and detection of dengue fever in China using Weibo messages [29]; another study analyzed Twitter data and predicted the peak of the incidence of COVID-19 in Canada using various symptom keywords such as *cough*, *runny nose*, and *anosmia* [30]. Using social media was also recognized to detect mental illnesses; according to Guntuku et al [31], depression and other mental illnesses were detected based on screening surveys, public sharing of a diagnosis, and patterns in languages and web-based activities via Twitter, Facebook, and web forums.

### Objective

With all that mentioned, this study aimed to gain a better understanding of digital dementia through a comprehensive analysis of Twitter data. The researchers used the hashtag #digitaldementia to conduct content and sentiment analysis, as Twitter is a popular social media platform with over 230 million monthly active users and 500 million daily tweets on average worldwide [32]. Twitter is recognized as a valuable source for monitoring public opinion on various health-related issues, including mental health [33-35].

## Methods

### Study Design and Data Collection

An exploratory content and sentiment analysis was performed. This study focused on a particular group of hashtags that had been used in the past to discuss the issue of digital dementia, including #digitaldementia, #digitalamnesia, #cyberdementia, and #digitalalzheimer. The study collected all tweets that contained at least 1 of these hashtags from January 1, 2008, to December 31, 2022, using Twitter’s application programming interface and Python packages (*Tweepy*) [36]. The raw data collected in this study include the author’s username, tweet content, time stamp, and any mentions or replies. The individual users were identified, and the user’s profile information was extracted through the appropriate application programming interface based on the collected data. All Twitter data collected and presented in this study were obtained in compliance with Twitter’s terms and conditions, which permit the use of publicly available content for syndication, broadcasting, distribution, retweeting, promotion, or publication, with the exception of personal information such as home addresses or identity documents [37]. It is important to note that no compensation was paid to the individual tweeters whose tweets were used in this study, per the agreement upon using Twitter.

### Inclusion and Exclusion Criteria

To create a data set of tweets related to digital dementia, publicly available tweets were searched in English or translated to English using Twitter’s automated translation feature. Using various combinations of hashtags with Twitter’s search function, we retrieved the most frequently hashtags, namely, #digitaldementia, #digitalamnesia, #cyberdementia, and #digitalalzheimer. These hashtags were coined by individuals who saw the excessive use of technology as a threat to human memory. To ensure the inclusion of tweets that specifically mentioned digital dementia without using the selected hashtags, the data set was expanded by searching for the hashtags as individual words. For instance, a search was performed for cases of “digital dementia” in addition to using the #digitaldementia hashtag.

At first, 33,498 tweets were collected using 4 specific search terms and imported into Microsoft Excel. After cleaning the data, 1964 (5.86%) tweets, written in the alphabet but not in English, were removed from the data set. Then, 884 (2.8%) non-retweeted duplicate posts were filtered out. In addition, tweets originating from social bots (ie, automated Twitter accounts) were detected and excluded to ensure an accurate representation of public discussions on digital dementia and related topics. Botometer was used to distinguish between genuine users and social bots; this tool assesses Twitter account characteristics and assigns a score to indicate the likelihood of an account being a bot, with a threshold set at ≥4 on a scale of 1 to 5 for English accounts [38,39]. All the accounts were screened after collection (not in real time). Out of the initial data set of 14,301 accounts, 1770 (12.38%) were identified as bots and removed. The analytical data set comprised 28,043 posts from 12,531 unique, verified, nonbot accounts after the comprehensive tweet cleansing process.

**Figure 1:**
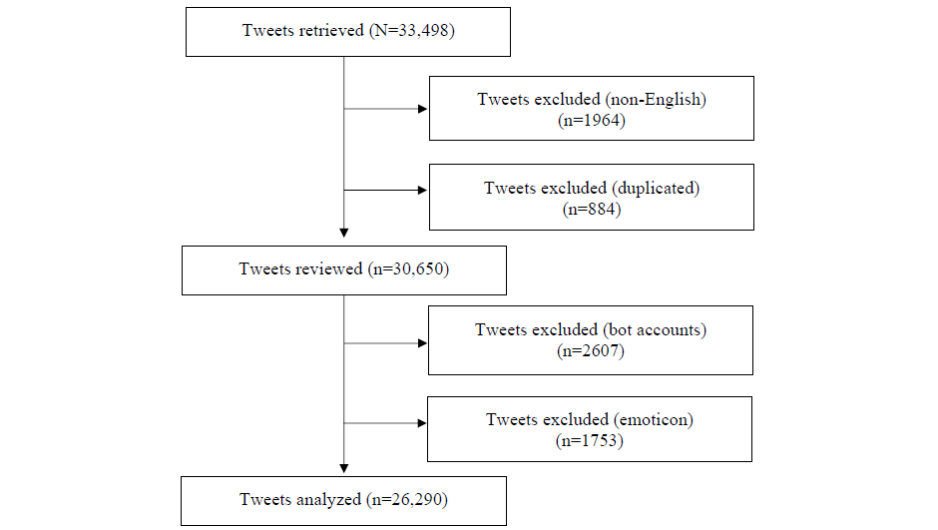
Flowchart of the inclusion and exclusion process.

The analysis excluded all tweets containing emoticons. This is because tweets often contain typos, ad hoc abbreviations, phonetic substitutions, ungrammatical structures, and emoticons, which can pose challenges for text-processing tools and introduce bias problems in sentiment analysis [40]. After removing tweets with emoticons, the final set for analysis comprised 26,290 tweets from 11,134 unique nonbot accounts. The inclusion and exclusion process of total tweets according to the workflow is detailed in Figure 1.

### Data Analysis of Tweets

Two different data analysis methods were used to achieve the research objectives: summative content analysis and sentiment analysis. The main goal of content analysis is to measure and explain unfamiliar phenomena [41]. This study used content analysis to identify and describe the textual elements of tweets related to digital dementia. In addition, the study used sentiment analysis, a technique used to analyze written language and determine the emotions conveyed in the text. This method is frequently used in health care and social media research to interpret textual information about the patient experience, with a focus on understanding the individual’s perspective [42].

Social media influencers are individuals who are considered opinion leaders and have the ability to influence the attitudes and behaviors of the audience [43]. Recent research has recognized the importance of studying the behavior of influencers on a particular issue to gain a better understanding of the societal context surrounding it [44]. On the basis of previous evidence highlighting the significant role of influencers in health care research [45], a content analysis was conducted on tweets authored by influencers among all the collected tweets. With that said, the aim of this study was to provide a more comprehensive insight into the current state of society’s context regarding the issue of digital dementia.

All collected tweets from the influencers underwent qualitative content analysis, a systematic approach for making inferences from text to summarize communication content [46]. This study considered 4109 tweets written by users with a minimum of 20,000 followers. On the basis of previous studies, these users were identified as social media influencers [47-49]. A codebook was developed for this study to use the inductive coding process that is widely used in Twitter content analysis [50]. The analysis followed several steps. Initially, to ensure reliability, raters reviewed a subset of 200 tweets from the influencer tweets (n=4109) to apply preliminary classifications to each category. Any differences in categorization and discrepancies among the evaluators were discussed until consensus was achieved, leading to adjustments in the classification criteria based on the initial ratings. Next, pairs of researchers, working independently, rated another second training set of 500 tweets from influencer tweets using the refined codebook. Cohen κ statistic was used to measure the interoperator reliability of the 500 tweets during the process. The Cohen κ metric spans from –1 to +1. If Cohen κ is ≤0, it signifies no agreement; if Cohen κ is between 0.6 and 0.8, it indicates strong agreement; and if Cohen κ is exactly 1, it represents perfect agreement [51]. The pooled Cohen κ statistic for 500 tweets independently coded by 2 researchers was found to be 0.84, representing excellent reliability.

Two researchers independently reviewed each tweet and manually coded the 3409 remaining tweets from influencers. In cases of classification discrepancies (<12%), the entire research team reviewed the tweet’s content and reached a final decision by a consensus of at least two-thirds of the members. Subsequently, 182 tweets with unclassifiable content were excluded. In total, 3927 influencer tweets were evaluated for content analysis.

The Valence Aware Dictionary and Sentiment Reasoner (VADER), a tool included in the *Natural Language Toolkit* library in Python, was used to analyze the overall sentiment of the collected tweets. VADER is a pretrained rule-based sentiment analysis system that evaluates sentiment by examining the words in a text. The toolkit operates based on a lexicon, where independent human evaluators have assigned ratings indicating each word’s positive or negative connotations. VADER calculates a composite sentiment score for each tweet by identifying and assessing words in the lexicon. It uses an algorithm that considers punctuation, capitalization, emoticons, and word modifiers to generate a sentiment score ranging from –1 to +1; a score of+1 signifies the highest level of positive sentiment, while –1 indicates the highest level of negative sentiment [52].

Before calculating the sentiment scores, all tweets containing punctuation and emojis were removed during preprocessing. Before the sentiment analysis, the Python software version 3.11.3 (Python Software Foundation) was used for natural language processing (NLP). Emojis were stripped from all collected tweets before conducting NLP tasks to analyze plain texts only. Subsequently, *Natural Language Toolkit*, a Python package for NLP tasks, segmented each tweet into tokenized words, removing stop words with minimal analytic value. Following the NLP tasks, sentiment analysis for the collected tweets was performed.

After tokenizing the words, sentiment analysis was conducted on all 26,290 tweets in this study. The sentiments linked with each theme were evaluated using 6 codes for sentiment analysis (Table 1). Each tweet was given a sentiment score ranging from –1 to +1 and then placed into one of the 6 categories given by Glaser and Strauss [53]. The framework is commonly used in Twitter-based research focusing on sentiment analysis, as suggested by Glaser and Strauss [53,54].

**Table 1:** Six codes for sentiment analysis.

Code	Definition
No sentiment	Tweets in this category lack emotional language or distinctive punctuation and often focus solely on mentioning the topic without conveying clear positive or negative sentiments. For instance, a tweet simply asking about the symptoms of digital dementia, such as “What are the early signs of digital dementia?” would be classified under this category.
Wretched	Tweets categorized as “wretched” express overwhelmingly negative sentiments related to digital dementia, occasionally containing slightly positive terms. The tweets may convey frustration, dissatisfaction, or negative experiences associated with digital technology. An example tweet might be, “Digital dementia is ruining my life. Can’t remember anything without my phone!” Sentiment scores for this category range from –1 to –0.6.
Bad	“Bad” tweets predominantly feature negative phrases and words, often expressing disappointment or concerns about digital dementia. While there might be occasional positive statements, the negative feelings overshadow the statements. An example could be, “Digital dementia is terrifying. Losing cognitive function to screens is a nightmare.” Sentiment scores for this category range from >–0.6 to –0.2.
So-so	Tweets in the “so-so” category convey a mixed or neutral sentiment regarding digital dementia. Positive and negative statements may balance each other out, or the tweet may remain neutral overall. Even if there are more negative phrases, the positive ones use stronger language than the negative ones. An example tweet might be, “Learning more about digital dementia. It's concerning, but there are ways to mitigate risks.” Sentiment scores for this category range from >–0.2 to <0.2.
Swell	“Swell” tweets predominantly contain positive terms related to digital dementia, with occasional mild negative phrases. However, the positive expressions outweigh the negative ones. For example, “Research shows simple lifestyle changes can combat digital dementia. Encouraging news!” Sentiment scores for this category range from 0.2 to <0.6.
Great	Tweets categorized as “great” are entirely positive in tone and wording, expressing strong affirmative feelings about digital dementia without any complaints. Tweets in this category may contain minimal negative language but are predominantly filled with enthusiastic praise or optimism. An example tweet could be, “Exciting breakthroughs in digital dementia research! There's hope for prevention and treatment.” Sentiment scores for this category range from 0.6 to 1.

After the sentiment analysis for the total collected tweets, to determine if there were significant differences in sentiment scores among the different groups defined during the process of content analysis, a one-way ANOVA was conducted. The one-way ANOVA is an appropriate statistical method in this context for several reasons. First, the methodology allows for the comparison of the means of ≥3 independent groups, making it suitable for analyzing the sentiment scores across multiple themes and subthemes. Second, the observed differences in sentiment scores can be analyzed to determine statistical significance or the possibility of occurring by chance, which is crucial for validating the robustness of our sentiment analysis and ensuring that our findings are not merely artifacts of random variability [55]. Furthermore, ANOVA is particularly advantageous in handling the variation within and between groups, providing a comprehensive understanding of the data’s underlying structure. By quantifying the variances, ANOVA offers a clear picture of how sentiment varies across different categories, which is essential for our research’s objective of understanding the nuanced emotional responses associated with each theme. Given the multiple themes and subthemes in this study, it is imperative to pinpoint which groups exhibit significant differences in sentiment scores, enabling the researchers to draw more precise and actionable insights from the data.

### Ethical Considerations

No ethics review was sought because the study only explored the publicly available data on social media and did not conduct any experiments on human participants. However, any personal study data, user IDs, followers, retweets, links, and emoticons were deidentified during analysis.

## Results

### Overview

This study analyzed 26,290 tweets spanning a 15-year period from 2008 to 2022. The study involved 11,134 Twitter users, with female users representing the highest proportion (n=5600, 50.3%). The primary countries of the users were the United States, followed by the United Kingdom and Northern Ireland **(**Table 2).

**Table 2:** Demographics of total Twitter users.

Characteristics			Users (n=11,134), n (%)		Tweets (n=26,290), n (%)
**Sex**					
	Female	5600 (50.3)		14,012 (53.3)	
	Male	4365 (39.2)		8465 (32.2)	
	Unknown	1169 (10.5)		3812 (14.5)	
**Country**					
	The United States	5088 (45.7)		13,854 (52.7)	
	The United Kingdom and Northern Ireland	2973 (26.7)		4521 (17.2)	
	Canada	913 (8.2)		2655 (10.1)	
	Australia	857 (7.7)		1708 (6.5)	
	India	256 (2.3)		1498 (5.7)	
	France	212 (1.9)		289 (1.1)	
	Germany	178 (1.6)		236 (0.9)	
	New Zealand	167 (1.5)		197 (0.7)	
	Singapore	111 (1)		148 (0.6)	
	Hong Kong	89 (0.8)		155 (0.6)	
	Philippines	45 (0.4)		51 (0.2)	
	Unknown	245 (2.2)		978 (3.72)	

### Content Analysis

During the analysis of tweets by social media influencers, 4 main themes and 11 subthemes were identified using an inductive thematic analysis approach grounded in robust qualitative research principles [56]. The aim was to capture nuanced and meaningful insights from social media discourse. A codebook was developed based on preliminary classifications and refined through interrater reliability assessments to ensure consistent and reliable content categorization. To identify potential domains, the research team conducted an extensive literature review. The codebook was iteratively refined as the coding progressed to include emerging subthemes, ensuring that it captured the full spectrum of issues discussed in the tweets. This iterative refinement process aligns with best practices in qualitative research and ensures that the coding framework remains flexible and responsive to the data’s nuances [57].

After analyzing tweet content, four themes emerged regarding digital dementia: (1) cognitive decline, (2) digital dependency, (3) technology overload, and (4) coping strategies. A comprehensive breakdown of primary themes and subthemes can be found in Table 3.

The content analysis identified 1526 (38.9%) of the total 3927 tweets, related to the theme of cognitive decline. These tweets were further categorized into 3 subthemes: the experience of cognitive decline due to the use of digital devices; scientific information about a decrease in brain activity; and problems with poor memory, concentration, and creativity.

**Table 3:** Content analysis of main themes and subthemes for influencers’ tweets (n=3927).

Main themes and subthemes		Tweets, n (%)	Example of phrases or #hashtags
**Cognitive decline (n=1526, 38.86%)**			“#Digitaldementia is real. Are we trading our brainpower for convenience?” “My memory is fading, focus is fleeting, and creativity is dwindling #DigitalDementia #MemoryLoss”
	Experience of cognitive decline due to the use of digital devices	859 (56.29)	
	Scientific information about a decrease in brain activity	448 (29.36)	
	Problems with poor memory, concentration, and creativity	219 (14.35)	
**Digital dependency (n=1218, 31.02%)**			“Why is my productivity so tied to technology? #digitalalzheimer” “Taking more time on my phone. decrease in my attention span and productivity. #DigitalAmnesia”
	Issues that arise as a result of the increase in digital device use in reality	549 (45.07)	
	The dependency of using digital devices	411 (33.74)	
	Anxiety or frustration without digital devices	258 (21.18)	
**Technology overload** **(n=779, 19.84%)**			“Too much digital input, too little mental output. #brainstrain #digitaldementia”
	Issues about digital information overload and cognitive strain	496 (63.67)	
	Stress caused by excessive information exposure on digital media	283 (36.33)	
**Coping strategies** **(n=404, 10.29%)**			“Stop living in a digital dreamland and start experiencing the real world before your brain turns to mush #DigitalDementia”
	Recommendations for alternative activities	234 (57.92)	
	Strategies for reducing digital device use	135 (33.42)	
	Public support	35 (8.66)	

A large number of tweets, 859 (56.3%), shared personal experiences of cognitive decline linked to excessive use of digital devices. These observations are consistent with research linking prolonged screen time to negative cognitive outcomes [58]. The tweet users frequently expressed concerns about the impact of digital device use on cognitive abilities:

My brain feels like it’s in a fog after too much screen time. #DigitalDementia #BrainFog

Another prevalent subtheme was identified through tweets that disseminated scientific information concerning cognitive decline. These tweets often discussed research findings and professional insights about how prolonged use of digital devices can lead to reduced brain activity. For example, recent studies suggest that excessive screen time can impact the brain structures responsible for critical cognitive functions [59]:

Studies show that prolonged digital exposure can lead to a decline in cognitive function. #Neuroscience #DigitalDementia

The remaining 219 (14.4%) tweets discussed issues related to poor memory, concentration, and creativity. Users frequently mentioned struggling with memory retention, managing study time, and maintaining creative thinking and problem-solving skills. The subtheme was identified by grouping tweets that described specific cognitive difficulties and perceived links to digital device use:

I can’t remember anything these days, and my focus is shot. Too much tech is ruining my brain. #MemoryLoss #DigitalDementia

Of the total 3927 tweets, the content analysis found 1218 (31%) tweets related to the theme of digital dependency. Digital dependency was categorized into 3 subthemes: increased digital device use, reliance on digital devices, and anxiety or frustration experienced in the absence of digital devices.

The analysis revealed a significant subtheme related to the various issues stemming from the growing use of digital devices. Numerous users expressed concerns about productivity, which was closely tied to technology use, and described feeling helpless or dependent, hindering real-world efficiency. This tendency was evident in tweets where users reflected on the inability to perform tasks without digital assistance, expressing similar sentiments; a typical tweet read as follows:

I’m so tied to my phone for everything that it’s becoming a problem. How do we function without them? #TechDependency

One prominent subtheme revolved around the pervasive dependency on digital devices. Tweets within this category often described a compulsive need to use smartphones or computers, even when such use was detrimental to productivity or mental well-being. For example, users frequently noted spending more time on phones, leading to a noticeable decline in attention spans and overall productivity. In addition, the emotional tone of the tweets highlighted a common narrative of struggle and acknowledgment of the negative impacts of digital dependency. A representative tweet stated as follows:

I’ve noticed a drop in my productivity because I can’t put my phone down. It’s a serious issue. #ScreenTime #DigitalAmnesia

The remaining 258 (21.2%) tweets addressed the anxiety or frustration experienced in the absence of digital devices. The final subtheme identified was the anxiety or frustration users experienced without digital devices. Tweets in the third subtheme often reflected a deep-rooted fear of missing out or a sense of unease when disconnected from the digital world. For example, some users mentioned needing access to platforms such as Twitter, underscoring the dependency on social media for daily interactions and information:

Being without my phone for a few hours made me so anxious. It’s scary how dependent I’ve become. #TechAddiction #Anxiety #digitaldementia

Of the total 3927 tweets, 779 (19.8%) tweets related to the theme of technology overload were identified. The theme of technology overload was further categorized into 2 subthemes: issues related to digital information overload and cognitive strain and stress caused by excessive exposure to information through digital media.

The most prominent subtheme related to technology overload comprised 496 (63.7%) tweets. The first subtheme, focusing on issues regarding digital information overload and cognitive strain, reflects the overwhelming amount of digital information people encounter daily. Previous research has shown that excessive information can lead to mental overload, reducing individuals’ ability to process information effectively [60]. Numerous tweets expressed frustration with the sheer volume of data and difficulty discerning relevant information. For example, users frequently mentioned feeling overwhelmed by constant updates, notifications, and the pressure to stay informed:

My brain feels overloaded with all the digital info I consume daily. It’s exhausting. #DigitalOverload #BrainStrain

The second subtheme, which comprised 283 (36.3%) tweets, focused on the stress experienced due to excessive exposure to information on digital media. This subtheme captured the emotional and psychological toll of continuous exposure to digital content. It is well-documented in the literature that prolonged digital engagement can lead to significant stress and anxiety [61]. The tweets under this subtheme often mentioned feelings of anxiety, stress, and burnout associated with digital media use:

Scrolling through endless feeds is stressing me out. Too much information all the time. #InfoOverload #Stress

Furthermore, of the total 3927 tweets, the analysis identified 404 (10.3%) tweets related to coping strategies. The coping strategies section was divided into 3 subthemes: recommendations for alternative activities, strategies for reducing digital device use, and public support.

The largest subtheme within coping strategies, comprising 234 (57.9%) tweets, focused on recommending alternative activities to reduce dependence on digital devices. Influencers often suggested physical exercise, hobbies, creative pursuits, and social interactions as substitutes for digital engagement:

Spent the day hiking and felt so much better without my phone. Nature is the best therapy. #DigitalDetox #RealLife

The second subtheme, with 135 (33.5%) tweets, offered specific strategies for reducing digital device use. The tweets in this category provided practical advice for limiting screen time, such as setting time limits, using tracking apps, or designating device-free zones:

Try setting a daily limit on your screen time to help reduce digital dependency. It works! #ScreenTime #TechBalance

The remaining 35 (8.7%) tweets discussed public support for managing digital dependency and cognitive decline. This category of tweets emphasized the importance of community and social support in coping with challenges. Influencers highlighted the role of peer support, community resources, and collective activities in enhancing resilience:

Join a local support group to share tips and get help managing digital addiction. You’re not alone. #SupportGroup #DigitalHealth

### Sentiment Analysis

The sentiment analysis of the tweets gathered provided more insights into the emotional tone of the collected data in this research. Table 4 offers comprehensive information about the corresponding sentiments, including the words, phrases, or hashtags used in the collected tweets for this study.

On the basis of the classification given by Glaser and Strauss [53], most tweets (14,492/26,290, 55.12%) fell under the categories of wretched (purely negative) or bad (mostly negative). By contrast, a small proportion of tweets (n=3122, 11.86%) were classified as great (purely positive) or swell sentiment (mostly positive). Approximately 4.2% (n=1107) tweets were classified as so-so, meaning the text had a balanced mix of negativity and positivity. In addition, 9% (n=2366) of the tweets had no sentiments.

The descriptive statistics for a quantitative analysis was also conducted. The sentiment score variable, representing the sentiment score for each tweet, had 23,924 observations with a mean of around –0.197 (SD 0.360), indicating the spread of sentiment scores around the mean. The sentiment code variable, following the classification by Glaser and Strauss [53], also had 23,924 observations. The mean sentiment code was approximately 2.509 (SD 0.853). The estimated mean sentiment score (–0.197, SE 0.002). The 95% CI for the mean sentiment score was approximately –0.202 to –0.193. These results indicate a high level of certainty (95% CI) that the true population mean sentiment score falls within this range. Since the CI does not include 0, it suggests that the mean sentiment score substantially differs from neutral sentiment. The results suggest an overall negative sentiment, as shown by the mean sentiment score being significantly below 0 and the CI not including 0, which represents neutral sentiment. The detailed statistics provide a solid basis for understanding the sentiment distribution and its implications within the data set.

Sentiment analysis provided valuable insights into the emotions associated with the main themes. Table 5 presents the main themes, sentiments, and phrases or hashtags that reflect the tweeters’ experiences.

Sentiment analysis was performed on a total of 3927 tweets from influencers, revealing interesting patterns across 4 major themes. The theme “cognitive decline” accounted for 38.9% (n=1526) of the tweets and included discussions about brain health and cognitive function. Positive sentiments were found in a small fraction, with 2.7% (n=41) mentioning brain training and cognitive enhancement and 11.1% (n=169) discussing brain games, mindfulness, and yoga. A substantial portion (n=498, 32.6%) showed a moderate sentiment with terms such as brain supplements and sleep quality, while negative sentiments dominated, with 34.2% (n=522) mentioning issues such as sleep deprivation, poor diet, and depression. The most severe concerns, such as dementia and Alzheimer disease, were reflected in 10.4% (n=159) of the tweets, and 9% (n=137) of the mentions did not reflect any sentiment, focusing on brain function and neurological changes.

The analysis of tweets revealed that 31.02% (1218/3927) of the tweets reflected digital dependency, indicating a reliance on digital devices. Positive tweets were minimal, with only 1.8% (n=22) discussing detox and mindfulness, while 21.6% (n=263) showed a more favorable sentiment with mentions of support groups and regulation. Tweets with a “so-so” sentiment accounted for 25.9% (n=315) and included discussions on consumption and prevention. Negative sentiments were prominent, with 39.9% (n=486) mentioning addiction, withdrawal, and low productivity. In addition, 4.9% (n=60) of the tweets highlighted severe impacts such as isolation and harassment.

Technology overload accounted for 19.84% (779/3927) of the tweets and focused on the excessive use of technology. Within this category, 10.1% (n=79) of the tweets had a positive sentiment, discussing mindful use and moderation. In addition, 16% (n=125) of the tweets expressed favorable sentiments with terms similar to advancements and innovation. By contrast, 20.5% (n=160) of the tweets had a moderately negative tone, addressing distractions and social media. Furthermore, 29.7% (n=231) of the tweets conveyed negative sentiments such as digital fatigue and stress. Finally, 12.6% (n=98) of the tweets in this category highlighted severe negative impacts, including addiction and dehumanization.

Finally, coping strategies were mentioned in 10.29% (404/3927) of the tweets, discussing methods to manage stress and challenges. Positive tweets (n=28, 6.9%) referred to support and counseling, while 36.1% (n=146) expressed a more favorable sentiment toward meditation, yoga, and creative activities. Tweets with a so-so sentiment (n=65, 16.1%) included relaxation techniques and time management, whereas 21.8% (n=88) reflected harmful coping mechanisms such as substance use disorder and self-harm. The most serious concerns, such as suicide and hopelessness, were mentioned in 7.4% (n=30) of the tweets, and 11.6% (n=47) did not reflect any particular sentiment, discussing masks, sanitizers, and information changes.

**Table 4:** Sentiment analysis of total tweets (n=26,290; original theme: digital dementia).

Sentiment	Tweets, n (%)	Examples of words or phrase
Great	741 (2.82)	“wins award,” “life story,” “joy in patients,” “better outcomes,” and “meditation”
Swell	2381 (9.06)	“magic martin,” “dementia diagnosis,” “excellent outlook,” “healthy tech,” and “bright future”
So-so	6310 (24)	“digital dilemma,” “robotic technology,” “clock,” “day,” “rely on digital,” “too much technology,” “affected by,” “irreversible,” “digital research,” “research community,” “young people,” “consultation,” “assistive,” “neuroscience,” “short-term memory,” “cognitive abilities,” and “webinar”
Bad	13,385 (50.91)	“digital dementia: a modern plague,” “destroying memory,” “stress anxiety,” “mental ill,” “suffer,” “cause death,” “kill brain,” “stop multitask,” “note overload,” “AI destroy,” “young blind,” “destroying ability,” “neurobiologist warns,” “destroying brain,” reliant on devices,” and “loss”
Wretched	1107 (4.21)	“making us stupid,” “digital dementia s*cks,” “dementia,” “disorder,” “diseases,” Parkinson’s,” “Alzheimer,” “worst,” “enslaved by,” “bad,” “hate,” “disrupting mental health,” “children stupid,” and “tech overuse”
No sentiment	2366 (9)	“diagnosis changed,” “info,” “usual,” “identification,” “contemplate,” and “sum up”

**Table 5:** Sentiment analysis of the influencer tweets (n=3927).

Main themes and subthemes			Tweets, n (%)		Examples of words or #hashtags
**Cognitive decline (n=1526, 38.86%)**					
	Great	41 (2.69)		“brain training,” “cognitive enhancement,” and “improvement”	
	Swell	169 (11.07)		“brain games,” “mindfulness,” “yoga,” and “healthy”	
	So-so	498 (32.63)		“brain supplements,” “sleep quality,” “engagement,” and “therapy”	
	Bad	522 (34.21)		“sleep deprivation,” “smoking,” “sedentary,” “poor diet,” “depression,” “chronic,” “injury,” and “neurological disorder”	
	Wretched	159 (10.42)		“dementia,” “Alzheimer’s,” “Parkinson’s,” “Huntington’s disease,” “injury,” and “impairment”	
	No sentiment	137 (8.98)		“brain function,” “brain structure,” “neurological changes,” and “age”	
**Digital dependency** **(n=1218, 31.02%)**					
	Great	22 (1.81)		“detox,” “mindfulness,” and “balanced”	
	Swell	263 (21.59)		“digital,” “support groups,” “regulation,” “control,” “coverage,” and “ongoing research”	
	So-so	315 (25.86)		“consumption,” “habits,” “overuse,” “prevention,” “dependence treatment,” “literacy,” and “media impact”	
	Bad	486 (39.9)		“addiction,” “compulsive,” “withdrawal,” “loss,” “low productivity,” “divide,” and “inequality”	
	Wretched	60 (4.93)		“isolation,” “loneliness,” “dehumanization,” “harassment,” “abuse,” “fraud,” “crime,” and “monopoly”	
	No sentiment	72 (5.91)		“screen time,” “digital media,” “virtual reality,” “statistics,” “facts,” and “knowledge”	
**Technology overload (n=779, 19.84%)**					
	Great	79 (10.14)		“mindful use,” “balance,” and “moderation”	
	Swell	125 (16.05)		“Advancements,” “innovation,” “new devices,” and “smart homes”	
	So-so	160 (20.54)		“Technology,” “screen,” “distractions,” “attention span,” “social media,” “online learning,” and “virtual meetings”	
	Bad	231 (29.65)		“digital fatigue,” “burnout,” “stress,” “anxiety,” “depression,” “loneliness,” “trolls,” “fake news,” and “scams”	
	Wretched	98 (12.58)		“addiction,” “dependency,” “isolate,” “disrupt,” “dehumanization,” “harmful,” and “digital divide”	
	No sentiment	86 (11.04)		“electronic device,” “visualization,” “network,” “found,” and “daily”	
**Coping strategies** **(n=404, 10.29%)**					
	Great	28 (6.93)		“support,” “counseling,” “services,” “forums,” “help,” and “therapy”	
	Swell	146 (36.14)		“meditation,” “yoga,” “breath,” “creative,” “hobbies,” and “exercise”	
	So-so	65 (16.09)		“thinking,” “relaxation,” “techniques,” “time,” “map,” “behavioral,” and “peer”	
	Bad	88 (21.78)		“substance,” “abuse,” “binge eating,” “self-harm,” “avoidance,” and “procrastination”	
	Wretched	30 (7.43)		“suicide,” “destructive,” “hopelessness,” “helplessness,” and “desperation”	
	No sentiment	47 (11.63)		“masks and sanitizers,” “info changing,” “prove hypothesis,” and “CDC”^a^	

^a^CDC: Centers for Disease Control and Prevention.

To gather more detailed statistics about the selected tweets within each theme, a one-way ANOVA for each theme was conducted. The total number of observations for collected tweets decreased during the content analysis because we excluded the observations for “no sentiment” tweets, which were treated as missing values. Before conducting the ANOVA, Shapiro-Wilk tests were performed to assess the normality of sentiment scores within each group. The results revealed that the data for each theme did not significantly deviate from normality.

Table S1 in Multimedia Appendix 1 analyzed the sentiment scores for different themes, revealing varying perceptions among the themes. Overall, the total mean sentiment score across all themes was –0.1072 (SD 0.4276) from 3585 samples.

As shown in Table S2 in Multimedia Appendix 1, the ANOVA results indicate significant differences in mean sentiment scores among the themes. The data’s total variation was divided into 2 components: variation between groups and within groups.

The between-group variation was measured for this study; the between-group sum of squares (SS) was 15.5574, with 3 *df*, and the mean square (MS) for between-group variation was calculated as SS divided by *df*, resulting in 5.1858. The *F*-statistic, the ratio of the between-group MS to the within-group MS, was 29.03, and the associated *P* value (probability>*F*) of <.001 indicates statistically significant differences in mean sentiment scores among the themes. This significant between-groups variation suggests that the average sentiment scores are different across the themes, meaning that each theme was perceived differently by the public, reflecting distinct emotional responses to all themes.

The study measured the variation within groups, finding a within-group SS of 639.7236 with 3581 *df*. The within-group MS was calculated as 0.1786 (ie, SS divided by *df*). The total SS of 655.281 represents the overall variability of the data, including both between-group and within-group variations. The within-group variation indicates the extent of individual differences in sentiment scores within each theme, highlighting the wide variability of individual responses within each theme; it is important for understanding the spread and consistency of sentiments within each theme.

A Bartlett test for equal variances was performed to assess the homogeneity of variances among the different themes (*χ*^²^_3_=82.1265; *P*<.001). This indicates that the variances among the themes are not equal, suggesting heterogeneity of variances. The variability in responses differs across themes, possibly due to the varied perceptions of the population.

The Scheffé test for multiple comparisons revealed significant differences between specific themes (Table S3 in Multimedia Appendix 1). “Digital dependency” had a significantly different mean sentiment score from “cognitive decline” by 0.065066 (*P*=.002). “Technology overload” differed substantially from “cognitive decline” by 0.097944 (*P*<.001) but not from “digital dependency” (0.032878; *P*=.455). “Coping strategies” had significantly higher mean sentiment scores compared with “cognitive decline” by 0.223505 (*P*<.001), “digital dependency” by 0.158439 (*P*<.001), and “technology overload” by 0.125561 (*P*<.001). These results indicate that while “cognitive decline,” “digital dependency,” and “technology overload” are associated with negative sentiments, “coping strategies” evoke a more positive response, with significant differences among these themes in public perception.

## Discussion

### Principal Findings

This study’s purpose was to identify topics related to digital dementia by examining the content and sentiment of tweets. It aimed to provide insights that could help manage the excessive use of digital devices, which has been linked to early-onset dementia [62]. The analysis of tweets showed that digital dementia is generally perceived negatively. The study found that negative tweets were prevalent, especially those related to cognitive decline, with an overwhelmingly negative sentiment rate of almost 70%. Furthermore, it is worth noting that digital dementia is a prevalent term used among Twitter users, and 38.86% (1526/3927) of tweets gathered were associated with cognitive decline in this study, highlighting the recognition of digital dementia as an issue in contemporary society.

According to this study, 55.12% (14492/26290) of all the tweets expressed unfavorable emotions. In addition, negative feelings, which included cognitive decline, digital dependency, technology overload, and coping strategies, predominated across all main themes based on the content analysis. These findings highlight the widespread negative perception of digital dementia. Matthews et al [63] have highlighted the growing concern of digital dementia, where excessive screen time among millennials and Generation Z is leading to increasing symptoms of mild cognitive impairment and Alzheimer disease and related dementias (ADRD); the study also indicated that this phenomenon could result in significant societal and economic burdens on the already overburdened health care services, emphasizing the need for coping strategies from the public and private sectors to mitigate these medical threats [54].

On the basis of the findings from the tweets, users identified cognitive decline as the most prominent issue (1526/3972, 38.86%). In addition, users reported experiencing sleep disorders, depression, and neurological disorders. Excessive use of digital devices can lead to digital dementia, which negatively impacts cognitive function [64]. Individuals who excessively use digital devices may experience cognitive decline and significant negative changes in personality, mood, and social interaction and even exhibit psychiatric symptoms. Moreover, excessive digital device use may lead to poorer social functioning, disrupted self-care routines, higher anxiety scores, and increased sleep disturbances for those affected by ADRD [65,66]. A literature review on the effects of screen time on children and adolescents suggests that media type, duration, content, and after-dark use of mobile devices are highly associated with neurological adverse consequences [67].

Digital dependency and technology overload are also recognized as dilemmas. The analysis of the tweets from this study showed that 31.02% (1218/3927) of the users were concerned about being too dependent on digital devices, while 19.84% (779/3927) were worried about being overloaded with technology. Importantly, the users mentioned that addiction problems, low productivity, and depression could arise from using digital devices excessively. A study conducted in South Korea reported positive correlations between smartphone addiction levels and depression, and women were found to be more susceptible to smartphone addiction than men [68]. Another study indicated that excessive use of digital technology and work pressure lead to burnout in journalists. The study highlights the need for appropriate resources and rest to mitigate the effects of digital technology use [69]. These findings highlight the growing apprehension regarding the impact of digital device use on people’s daily lives. It is vital to thoroughly examine the advantages and disadvantages of using such devices and increase awareness of the associated risks in the near future.

Among the 4 main themes, coping strategies were emphasized. The results showed that engaging in alternative activities, reducing digital device use, and seeking public support could prevent digital dementia. Regarding sentiment analysis of the coping strategies, 36.1% (146/404) of the users expressed a positive sentiment, proposing participation in yoga, meditation, and exercise.

Regular physical activity can help prevent ADRD by increasing cognitive reserve [70]. Engaging in high-intensity training for at least 20 minutes on ≥3 days per week or participating in moderate-intensity training on ≥5 days per week is considered physical activity and positively impacts brain neurodegenerative diseases [71]. In addition, incorporating yoga and meditation interventions into the prevention process of dementia can be beneficial in preventing the onset of the condition. Modifying lifestyle factors such as dietary changes and physical activity through yoga and meditation intervention could be a cost-effective approach to managing dementia [72,73]. Wilmer et al [23] also indicated that the temporary cessation of smartphone use has positive effects, potentially alleviating digital fatigue. Recent research findings have shown that reducing the time spent using digital devices can significantly help prevent cognitive impairment and lessen the onset of digital dementia [74,75]. Several studies suggest reducing screen time can improve concentration, learning, and memory [76,77]. It can also enhance psychological well-being and reduce experiences of anxiety and depressive moods [78,79]. Furthermore, decreasing the use of digital devices can improve sleep quality and overall mental health [80]. Notably, there has been a global movement toward addressing digital dependency. World Digital Detox Day is observed to cope with the prolonged exposure to digital technologies while enhancing awareness of the negative effects of using digital devices [81]. Digital-free tourism has also become an emerging solution for family and social engagements [82]. A digital detox program in 2 preparatory governmental schools in Egypt indicated positive results; the high rate of screen addiction among students dropped to 14.3% in the posttest measures compared with 20% in the pretest measures [83].

### Strengths and Limitations

This study explored the reactions and perceptions of digital dementia among Twitter users, taking a comprehensive approach to vividly portray users’ attitudes toward digital dementia. Using Twitter as the data source facilitated a compelling exploration of public sentiment. The methodology used enabled the creation of a sizable data set, which is difficult to achieve through traditional survey methods. Importantly, the authors believe this is the first study seeking public perception of digital dementia via Twitter.

There are several limitations to note. First, it is important to note that the study’s results may not accurately represent the entire population’s views on digital dementia due to the high concentration of Twitter users [84]. Thus, it is crucial to understand that the opinions expressed in the study reflect only a subset of individuals rather than the entire population’s views.

Although the study extracted a large amount of data, only Twitter was used for this study. Other social media platforms could provide more information regarding digital dementia. Twitter limits users to 140 characters per tweet, restricting users from fully expressing opinions or sentiments. This constraint raises concerns about the comprehensiveness of user contributions. For instance, there may be more 2-way communication on social networking sites such as Facebook, which may differ from that of Twitter [85]. As the proposed hashtags are also available on other social media platforms, such as Facebook and Instagram, future research could explore whether engagement of users differs from that of Twitter.

Previous research has shown a higher proportion of Twitter users in urban areas compared with rural areas, which could introduce biases [86]. Furthermore, Twitter is overrepresented by a younger population aged 25 to 34 years compared with the general population [87].

In addition, the analysis was restricted to English-language tweets; insights from other languages could provide valuable perspectives. Finally, tweets containing emojis were excluded from this study’s NLP task, having a sufficient number of tweets to carry out sentiment and topic analysis. However, incorporating emojis could have provided valuable insights into users’ emotions, resulting in a more nuanced sentiment analysis. That said, future studies should consider analyzing emojis based on a shorter period.

### Conclusions

The study emphasizes the various concerns associated with digital dementia via Twitter. By exploring the content and sentiment of the recent tweets regarding digital dementia, the findings suggest that the perception of digital device use is increasingly negative, with rising concerns about its effects on mental health. Moreover, individuals are worrisome of digital dependency and technology overload in everyday lives. To address these concerns effectively, it is necessary to implement a range of coping strategies, such as yoga, exercise, and meditation, to prevent early-onset dementia. The results of this study could serve as the foundation for those performing research regarding digital dementia and for the betterment of the dementia community.

## Appendix

Multimedia Appendix 1 Sentiment score by theme, ANOVA, and comparison of sentiment score by theme (Scheffé).

Multimedia Appendix 2 Raw data used for content analysis.

## Abbreviations

ADRD: Alzheimer disease and related dementias
MS: mean square
NLP: natural language processing
SS: sum of squares
VADER: Valence Aware Dictionary and Sentiment Reasoner

## Data Availability

The raw data for content analysis can be found in Multimedia Appendix 2, and the principal codes for data sets generated and analyzed during this study are available from GitHub [88].
