# Supplementary material for: Twitter Discussions on #digitaldementia: Content and Sentiment Analysis
Source: J Med Internet Res. 2024 Jul 16;26:e59546. doi: 10.2196/59546 (PMC11289583; doi:10.2196/59546)
Supplement: Multimedia Appendix 1 [file jmir_v26i1e59546_app1.docx]

| **Table S1**. Sentiment score by theme | | | |
| --- | --- | --- | --- |
| Theme | Mean  Sentiment score | Std. Dev. | Frequency |
| Cognitive Decline | -0.1692 | 0.3944 | 1,389 |
| Digital Dependency | -0.1041 | 0.3883 | 1,146 |
| Technology Overload | -0.0713 | 0.5039 | 693 |
| Coping Strategies | 0.0543 | 0.4606 | 357 |
| Total | -0.1072 | 0.4276 | 3,585 |

| **Table S2**. Analysis of variance | | | | | | | | | |
| --- | --- | --- | --- | --- | --- | --- | --- | --- | --- |
| Source | | SS | | df | MS | | F | | Prob > F |
| Between Groups | | 15.5574 | | 3 | | 5.1858 | 29.03 | | 0 |
| Within Groups | | 639.7236 | | 3581 | | 0.1786 |  | |  |
| Total | | 655.281 | | 3584 | | 0.1828 |  | |  |
| Bartlett's equal-variances test: chi-square(3) = 82.1265  Prob>chi-square = 0.000 | | | | | | | | | |
| **Table S3**. Comparison of sentiment score by theme (Scheffe) | | | | | | | |  |  |
| Row Mean- Col Mean | Cognitive Decline | | Digital Dependency | | Technology Overload | | |  |  |
| Digital Dependency | 0.065066 (0.002) | |  | |  | | |  |  |
| Technology Overload | 0.097944 (0.000) | | 0.032878 (0.455) | |  | | |  |  |
| Coping Strategies | 0.223505 (0.000) | | 0.158439 (0.000) | | 0.125561 (0.000) | | |  |  |
